# Standardization of techniques used in facial nerve section and facial movement evaluation in rats

**DOI:** 10.1016/S1808-8694(15)30966-6

**Published:** 2015-10-19

**Authors:** Simone Damasceno de Faria, José Ricardo Gurgel Testa, Andrei Borin, Ronaldo N. Toledo

**Affiliations:** a MD, otorhinolaryngologist, MS student - ORL-UNIFESP; b PhD in Medicine, MS - UNIFESP, Associate Professor of Pediatric Otorhinolaryngologist - UNIFESP-EPM, PhD student - UNIFESP; c MS in Sciences - UNIFESP, PhD student; d MS in Sciences - UNIFESP, PhD student. Universidade Federal de São Paulo – Federal University of São Paulo

**Keywords:** evaluation, surgery, facial nerve, facial palsy, rats

## Abstract

**Aim:**

standardization of the technique to section the extratemporal facial nerve in rats and creation of a scale to evaluate facial movements in these animals before and after surgery.

**Study design:**

Experimental.

**Method:**

twenty Wistar rats were anesthetized with ketamine xylazine and submitted to sectioning of the facial nerve near its emergence through the mastoid foramen. Eye closure and blinking reflex, vibrissae movement and positioning were observed in all animals and a scale to evaluate these parameters was then created.

**Results:**

The facial nerve trunk was found between the tendinous margin of the clavotrapezius muscle and the auricular cartilage. The trunk was proximally sectioned as it exits the mastoid foramen and the stumps were sutured with a 9-0-nylon thread. An evaluation and graduation scale of facial movements, independent for eye and vibrissae, was elaborated, together with a sum of the parameters, as a means to evaluate facial palsy. Absence of eye blinking and closure scored 1; the presence of orbicular muscle contraction, without blinking reflex, scored 2; 50% of eye closure through blinking reflex, scored 3, 75% of closure scored 4. The presence of complete eye closure and blinking reflex scored 5. The absence of movement and posterior position of the vibrissae scored 1; slight shivering and posterior position scored 2; greater shivering and posterior position, scored 3 and normal movement with posterior position, scored 4; symmetrical movement of he vibrissae, with anterior position, scored 5.

**Conclusion:**

The rat anatomy allows easy access to the extratemporal facial nerve, allowing its sectioning and standardized suture. It was also possible to establish an evaluation and graduation scale of the rat facial movements with facial palsy based on the clinical observation of these animals.

## INTRODUCTION

Facial paralysis caused by facial nerve injury is a relatively common clinical condition. Its unilateral manifestation may cause alterations in facial symmetry, incomplete eye closure with or without ophthalmic repercussion, difficulty swallowing and in pronouncing some phonemes, amongst other problems. Therefore, this condition brings about relevant social and individual consequences, requiring great efforts in the attempt to understand the many factors related to facial nerve injury, its regeneration and repair, so as to reduce its consequences.

The facial nerve motor fibers, although they originate in the central nervous system, have great regenerative capacity, comparable to a peripheral motor nerve. The facial nerve has a debris and myelin clearance greater than that of the Central Nervous System (CNS) and, contrary to what happens to the CNS, the facial nerve has some sort of guidance for its regenerative process. The facial nerve also has better response capability to growth factors after injuries^1^.

After injury, the axon is influenced by a number of events, among them we list: local paracrine factors, retrograde molecule transportation (cytokines and growth factors), calcium and sodium inflow, generation of free radicals, increase in the activity of phospholipases, generation of hyaluronic acid derivatives, genetic factors (balance between bcl2 and bax/bak)^1^.

Wallerian degeneration, which follows nerve injury, is influenced by these factors and is completed in approximately thirty days, causing the removal of the distal axon fragment. In the regeneration process, which starts in the first Ranvier node, close to the injury site and that has a growth rate of one to two millimeters per day, the Schwann cells play a fundamental role (they remove neural debris, secrete cytokines and growth factors); notwithstanding, the integrity of the endoneurium basal membrane is necessary in order to guide this process^1^.

Thus, the type of injury to which the nerve is submitted is of fundamental importance to the regeneration process and the approach to be used. Sunderland classified the nerve injury based on histopathological findings into: first grade – intact nerve structure (neurapraxia); second grade – ruptured axon with intact Schwann cells (axonotmesis); third grade injury to the endoneurium; fourth grade – injury to the perineurium and fifth grade – complete rupture (neurotmesis). The first two injury grades usually allow full nerve recovery^1^. However, when there is loss of nerve continuity, its direct repair has been advocated as the most effective way to specifically rehabilitate the facial nerve. When this is not possible, it is necessary to use nerve grafts^2^.

Although surgical techniques have improved, the functional result related to the repair of full injuries to the facial nerve is still short of being satisfactory^1^, ^2^. Such fact has fostered may experimental studies in attempts to establish the best approach^2^, ^3^, ^4^, and the best moment to approach an injury to this nerve^5^, ^6^, ^7^, ^8^.

Another line of research is to study the substances that act on facial nerve regeneration after injury, in an attempt to introduce a therapeutic alternative that may bring about better results, specially in paralysis caused by a full section of the facial nerve. Among these substances we have the neurotrophic factors^9^, androgens^10^ and nimodipine^11^.

With the progress we are seeing in experimental research, many animal models have been used (rats, mice, rabbits, cats). However, there is no experimental model standardized to study facial paralysis – behavioral evaluation, approach technique, facial nerve injury and regenerative process onset.

It is of great importance to establish a scale to assess facial movements and to standardize the surgical technique used to approach the facial nerve and the various stages of its regenerative process in order to better develop investigations on systemic drugs that may promote a better recovery for the facial nerve.

The goal of the present study is to standardize the technique of sectioning the facial nerve extratemporally in rats and establish a scale to evaluate facial movement in these animals before and after the sectioning of this cranial nerve.

## MATERIALS AND METHODS

Twenty adult, male, Wistar rats, weighing between 250 and 300g, were used in this study. The animals were kept in a proper room, with light-dark cycles of 12 hours. Housed in proper cages, they had free access to food and water. All the procedures were carried out according to the recommendations from the Ethics Committee in Research of the São Paulo Hospital – Federal University of São Paulo (UNIFESP)

These rats were anesthetized by intraperitoneal injection of 2% Xylazine (0.5ml/kg) and 10% ketamine (0.9ml/kg). After that, the hair from the right side retroauricular region was removed. They were then placed in lateral decubitus and received an injection of xylocaine (it was repeated if necessary) in this region, and then we started the surgical procedure, which resulted in right side peripheral facial paralysis in these animals. The procedure was carried out by two surgeons, through the use of surgical microscopes DF-Vasconcellos M90, with a 200mm lens and 16x magnification. Surgical results were photographed with a 3.2 megapixels Sony Cybershot camera and were standardized.

All the animals were observed as to spontaneous and stimulated facial movements. This assessment was carried out by the same observer, and occurred before the surgery (to rule out facial movement alterations present prior to surgery that could compromise the later assessment), and in alternate days in the postoperative time, during 30 days. The parameters observed were: eye closure, blinking reflex, vibrissae movement and positioning. To do that, each rat was individually evaluated in a cardboard box (37×21×13cm) painted black inside (to better visualize the vibrissae), and as stimulation we used air inflation (a 20 ml syringe) on the animal face to trigger the blinking reflex, and hand clapping (three to four times) to cause vibrissae movement. The left side served as the control side (blinking reflex present with complete eye closure; normal and correctly positioned vibrissae movement). Through this observation we created a scale to assess facial movement in these animals.

## RESULTS

With the animals properly positioned, the surgery started. After incision in the right retroauricular region (about three centimeters), the surgeons proceeded with the dissection by planes. The platysma muscle was superficially identified, and its cranial portion was sectioned. The tendinous border of the clavotrapezius muscle was found at the postero-superior portion of the incision and the facial nerve trunk was located close to this border, which was then pushed posteriorly in order to better show the nerve emergence. There was no need to cut this border. The nerve trunk was then followed antero-inferiorly down to its furcation, located medially to the external jugular vein and its branches. The facial nerve branches covered by fascia were not dissected.

The facial nerve trunk was then cut with the scalpel, proximal to its emergence, and then sutured with nylon 9.0 wire. The stumps were sutured with one stitch. In seven animals (35%), a small branch came off the main trunk close to the mastoid foramen. In four cases, we sectioned and sutured the branch and the trunk, however in the other three cases we managed to isolate the main trunk and section it alone.

After epineurium suture, we closed the skin with simple stitches made by nylon 4-0 wires, cut close to the incision (Pictures 1 to 4)

No animal was excluded from the study. Based on our observation after nerve cutting and suturing, it was possible to establish a scale to assess facial movements in these rats. Eye closure through the blinking reflex and vibrissae movements were observed and distributed separately in one scale.

Vibrissae movement and its anterior positioning, symmetrically in the control side, was established as a sign of complete functional recovery of this facial nerve branch in the operated side, and received a score of 5. Blinking reflex present with complete eye closure also received a score of 5 points. Therefore, the complete recovery of the right side of the face received a score of 10, same value assigned to facial movements before surgery (complete recovery was not seen in any animal for 30 days).

The complete lack of blinking reflex without ocular closure scored 1 point, and the lack of vibrissae movements with posterior positioning scored 1 point as well. Thus, complete facial paralysis scored 2 and was seen in all rats that underwent the surgical procedure.

A light vibrissae tremor on the anterior position, scored 2 points, tremor with the vibrissae positioned more posteriorly scored 3 and the normal vibrissae movement keeping its posterior position scored 4 points.

The lack of blinking reflex (no ocular closure) and the contraction of the orbicular muscle scored 2 points; the closure of half the ocular cleft (50%) with blinking most are nerve sectioning and nerve crushing. However, there is no established pattern for facial nerve surgical approaches and for the observation of the subsequent facial paralysis in these animals. This standardization could bring great uniformity to the studies with certain animals and would certainly facilitate result comparisons.

**Figure 1:**
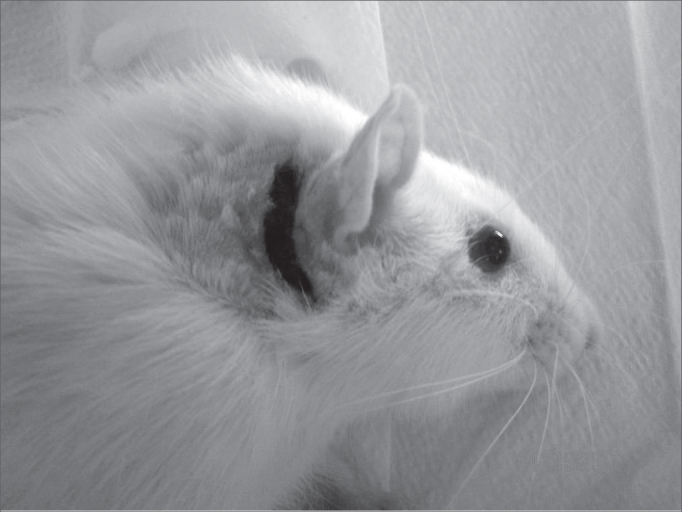
Retroauricular incision in the skin and subcutaneous tissue.

**Figure 2:**
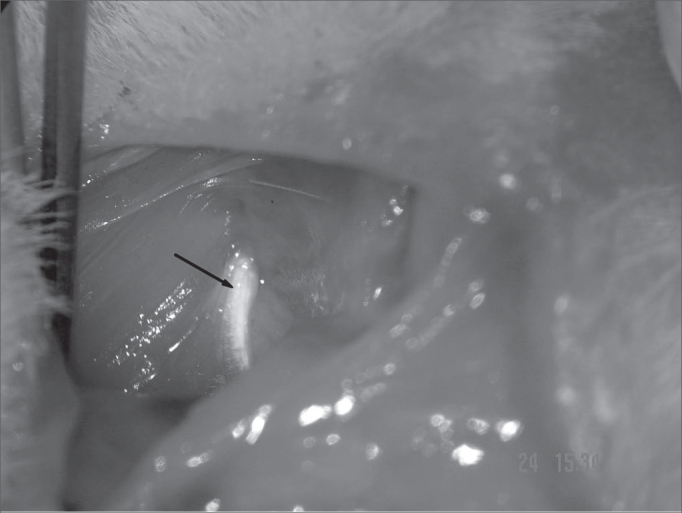
Tendon border of the clavotrapezius muscle- arrow.

**Figure 3:**
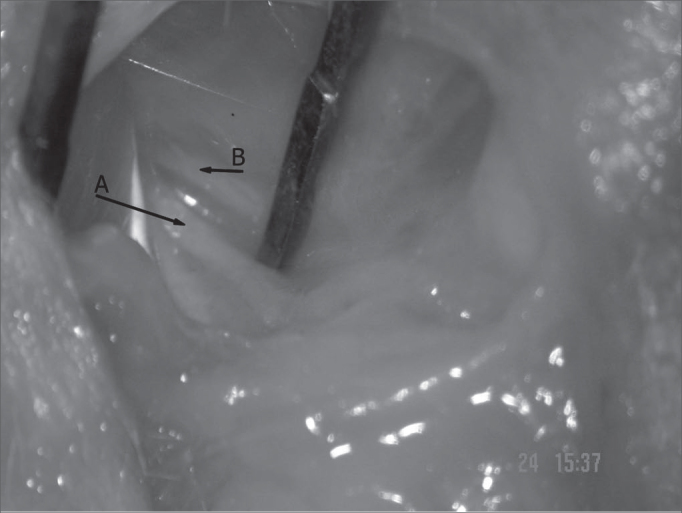
Facial nerve trunk: main trunk - arrow A; posterior auricular branch - arrow B.

**Figure 4:**
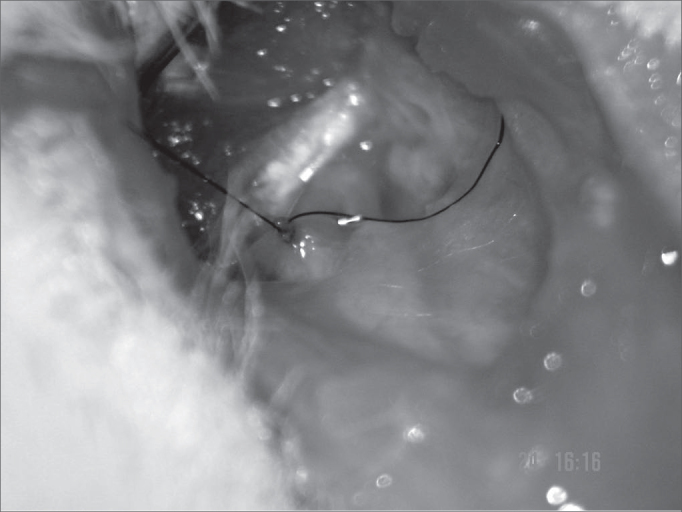
Approximating the facial nerve stumps. nylon 9-0.

**Figure 5:**
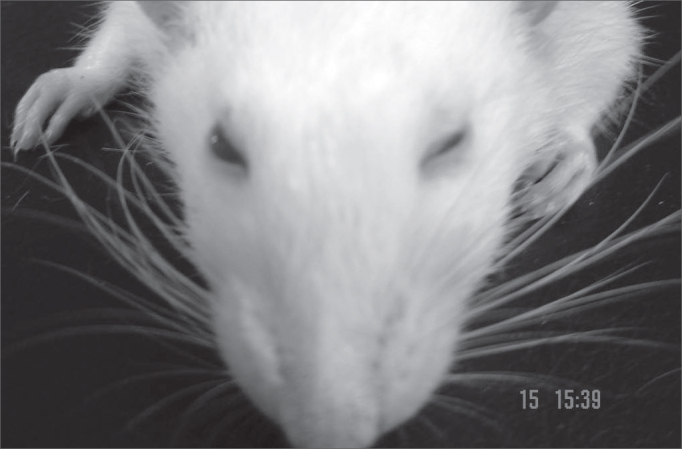
Evaluation under stimulus in the immediate post-op: blinking reflex present with complete eye closure and vibrissae movement on the anterior position on the left side. To the right side: no eye movement and no vibrissae movement, they are posteriorly positioned (1 point for the vibrissae and 1 for the eyes – right side).

**Figure 6:**
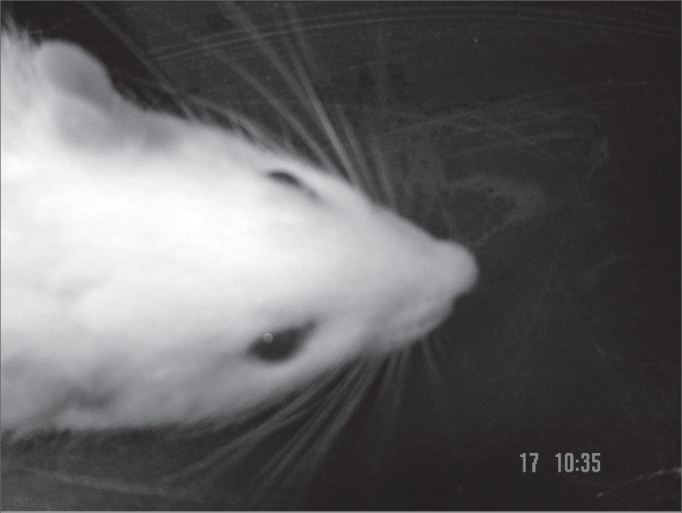
Spontaneous evaluation before sectioning the facial nerve – vibrissae showing symmetrical movements and anterior position (5 points).

**Figure 7:**
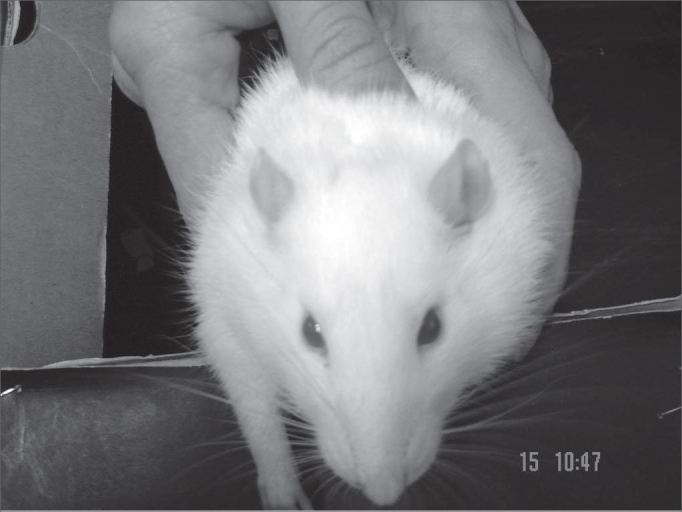
Animal presenting normal vibrissae movement and anterior position, to the left (five points); greater tremor on the vibrissae and posterior position, to the right (3 points).

A large number of studies have used rats as experimental models because they are easy to acquire and they tolerate well the facial paralysis, even when bilateral, without presenting problems in the immediate postoperative time^13^.

The rats used in the present investigation were the Wistar which, together with the Sprague-Dawley, are the most frequently used.

The animals were anesthetized by intraperitoneal reflex present scored 3 points; and the almost total closure (75%) scored 4 points.

**Chart 1:**
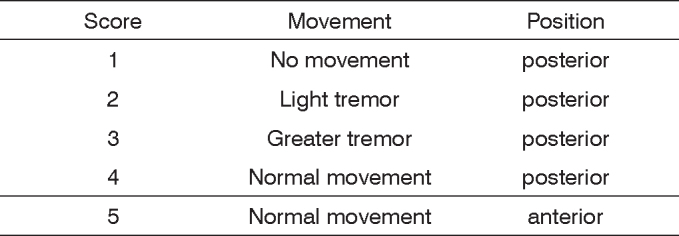
Vibrissae observation scale.

**Chart 2:**
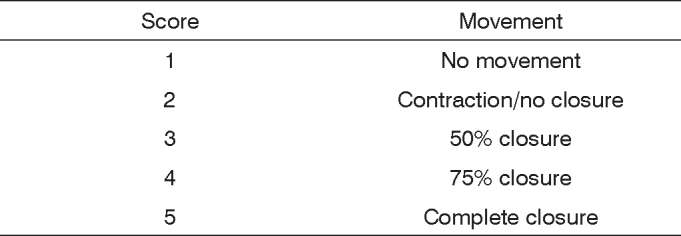
Scale of eye closing and blinking reflex observation.

Charts 1 and 2 show the scales created:

The animal assessment through this scale may be exemplified in the pictures that follow. (Pictures 5 to 7).

## DISCUSSION

The human face may be considered a mirror of our feelings, since it is through the movement of its muscles that we express different types of feelings in different degrees. It also has a fundamental participation in social communication because it is integrated to the speech process.

Facial nerve integrity, responsible for the innervation of the facial muscles, bears a relevant role in the social and individual context. Important physiological functions depend on this integrity, such as tearing and ocular protection, taste (anterior 2/3 of the tongue), food intake (mouth orbicular muscle takes part in the initial process), salivation, among others^12^. Many are the causes of this dysfunction, inflammatory processes and injuries are among the most common.

Because of the relevant impact its dysfunction may cause, the facial nerve has been studied in the most varied ways, and experimental studies play a key role in this process.

Many animals have been used as experimental models, among them we list: rabbits, pigs, rats, mice and cats. Different types of facial nerve injuries are described in facial paralysis investigations, and the two that appear the injection of 2% Xylazine (0.5ml/kg) and 10% ketamine (0.9ml/kg). Such combination is often used to anesthetize both rats and other animals used in experimental studies2,7,14-16.

The retroauricular incision used in this study allowed a clear field of vision to explore the facial nerve, and it is frequently considered a reference in papers that describe this approach in rats^13^, ^14^ in details and in other animals^12^. Another very much used incision is the infraauricular^17^, ^18^.

The facial nerve may be approached in its intratemporal pathway^6^, ^12^, ^14^, notwithstanding; we chose to approach it in its extratemporal pathway, close to its emergence through the mastoid foramen, because it is the most commonly used way in studies that cause facial paralysis in rats^2^, ^3^, ^16^, ^19^, ^20^, ^21^.

In order to find the facial nerve emerging through the mastoid foramen, we sectioned the platysma muscle and used the auricular cartilage and the tendon border of the clavotrapezius^13^, ^14^ muscle as reference points. This way, we located the main trunk of the facial nerve, between the auricular cartilage, anteriorly, and the clavotrapezius posteriorly. We followed this trunk until we found its furcation into minor branches, in order to confirm it that structure was really the facial nerve, since we did not use intraoperative stimulation on the nerve. The use of intraoperative stimulation on the facial nerve has been described only in a handful of studies^2^, ^18^, ^19^.

Having found the nerve trunk, we sectioned it close to the mastoid foramen. However, in seven animals the posterior cervical branch, probably, diverged from the main trunk still close to the mastoid foramen, was difficult to isolate and section. In four cases we sectioned the trunk and the branch, and in the other three, we were able to isolate the main branch and cut it. We did not find references in these regards in the literature. After sectioning the trunk, the stumps were sutured with a single epineurium stitch, using a nylon 9-0 wire because it is technically easier. Although the stump suturing was been carried out by Fernandez et al. (1992 and 1995)^3^, ^22^ with a nylon 10-0 wire and by Guntinas-Lichius et al. (2001 and 2005)^16^, ^21^ with nylon 11-0 wire, Yian et al. (2001)15 in his studies with rabbits used the nylon 8-0 suture wire.

And finally we sutured the skin with simple nylon 4-0 stitches. Fernandez et al. (1995)^22^ used metal clips to close the skin, while Bento & Miniti (1989)^12^ in their study with cats, used catgut 2-0 wire to close the skin in a single layer; and Mersa et al. (2000)^2^ used separate nylon 4-0 stitches to close the rabbit skins. This final step of the surgical procedure also varies and is also not very often mentioned in the literature.

Observing the behavior of the animals involved in the facial paralysis study has been used as a parameter to assess facial nerve regeneration. Studies with rabbits^15^, hamsters^10^ or rats^2^, ^14^, ^20^ observed eye closure, blinking reflex and vibrissae movement and positioning. In non-operated animals, the blinking reflex should be present as symmetrical and complete eye closure, as well as vibrissae movement, which should be positioned anteriorly. Thus, in animals with facial paralysis, there are characteristic signs that include the loss of the blinking reflex and no vibrissae movement, which remain posteriorly positioned^10^, ^20^, ^21^. By observing these signs, distinct facial paralysis scales have been described.

Yian et al. (2001)^15^ developed a scale to observe upper lip and eyelid movements in rabbits (who underwent cutting of facial nerve branches), with and without stimuli (forehead and nose touch). This scale assigned values between 0 and 3, 0 was assigned to the lack of movement and 3 to normal movement; 1 and 2 points were given to mild and moderate movement, respectively. Byers et al. (1998)^14^ also described a subjective observation scale for rats, after cutting their facial nerve at its canal, considering the parameters mentioned above. This scale varied from 0 (no movement) to 100% (complete movement) and, like the other, approached eyes and vibrissae together. Gilad et al. (1996)^20^ assessed only the vibrissae function, their normal movement and position. In their study, Mersa et al. (2000)^2^ observed eye closure and the blinking reflex, and they built a scale with values between 0 and 2; and 0.5 was assigned only to the contraction of the orbicular muscle contraction. In their study with cats, Bento & Miniti (1989)^12^ studied behavior, paying special attention to eye closure and the blinking reflex, and the presence or absence of facial movement in a symmetrical fashion. Haldlock et al. (2005)^18^ created a scale with values between 0 and 1, but contrary to these studies, they applied it separately to assess vibrissae movement and eye closure. They justified this separation based on eye and vibrissae function recovery, which occurred at distinct times.

We have then created an observational scale in order to assess vibrissae position and movement, and another, with the same variation, to assess eye closure and blinking, because we noticed the asymmetrical recovery of facial function as mentioned by Haldlock et al. (2005)^18^. The parameters assessed were similar to the ones mentioned: lack of movement, vibrissae tremor, normal movement and their posterior or anterior positioning; contraction of eye orbicular muscles, complete and incomplete eye closure. The scale has five scores (between 1 and 5), and also a separate score for the parameters. It includes a summation of points for each distinct parameter, similar to the Facial Gradation System presented by Ross et al.^23^ in 1996 to clinically assess facial recovery in human beings. Thus, it is possible to have an idea of the recovery for one isolate parameter and for the whole face of the animal, and also to compare animals from different groups (if the study involves both a study group and a control group), in a more accurate fashion.

Therefore, the rat allows the observation of its eyes and vibrissae movements and also the creation of an independent scale that allows a better analysis of these parameters and of facial movements as a whole. The facial nerve approach, in its extratemporal pathway, with cutting and suturing, is also a procedure that may be standardized in the rat, because this animal has an accessible facial nerve trunk and with such variability that does not compromise such approach.
